# Cloning and characterization of *KoOsmotin* from mangrove plant *Kandelia obovata* under cold stress

**DOI:** 10.1186/s12870-020-02746-0

**Published:** 2021-01-06

**Authors:** Jiao Fei, You-shao Wang, Hao Cheng, Yu-bin Su, Yongjia Zhong, Lei Zheng

**Affiliations:** 1 State Key Laboratory of Tropical Oceanography, South China Sea Institute of Oceanology, Chinese Academy of Sciences, Guangzhou, 510301 China; 2 Southern Marine Science and Engineering Guangdong Laboratory, Guangzhou, 511458 China; 3 Innovation Academy of South China Sea Ecology and Environmental Engineering, Chinese Academy of Sciences, Guangzhou, 510301 China; 4 College of Life Science and Technology, Jinan University, Guangzhou, 510632 China; 5 Root Biology Center, Fujian Agriculture and Forestry University, Fuzhou, 350002 China

**Keywords:** Osmotin, Mangrove plant, *Kandelia obovata*, 3D model, Gene expression, Cold-resistance

## Abstract

**Background:**

Low temperature is a major abiotic stress that seriously limits mangrove productivity and distribution. *Kandelia obovata* is the most cold-resistance specie in mangrove plants, but little is known about the molecular mechanism underlying its resistance to cold. Osmotin is a key protein associated with abiotic and biotic stress response in plants but no information about this gene in *K. obovata* was reported.

**Results:**

In this study, a cDNA sequence encoding osmotin, *KoOsmotin* (GenBank accession no. KP267758), was cloned from mangrove plant *K. obovata*. The KoOsmotin protein was composed of 221 amino acids and showed a calculated molecular mass of 24.11 kDa with p*I* 4.92. The KoOsmotin contained sixteen cysteine residues and an N-terminal signal peptide, which were common signatures to most osmotins and pathogenesis-related 5 proteins. The three-dimensional (3D) model of KoOsmotin, contained one *α*-helix and eleven *β*-strands, was formed by three characteristic domains. Database comparisons of the KoOsmotin showed the closest identity (55.75%) with the osmotin 34 from *Theobroma cacao*. The phylogenetic tree also revealed that the KoOsmotin was clustered in the branch of osmotin/OLP (osmotin-like protien). The KoOsmotin protein was proved to be localized to both the plasma membrane and cytoplasm by the subcellular localization analysis. Gene expression showed that the *KoOsmotin* was induced primarily and highly in the leaves of *K. obovata*, but less abundantly in stems and roots. The overexpressing of *KoOsmotin* conferred cold tolerance in *Escherichia coli* cells.

**Conclusion:**

As we known, this is the first study to explore the osmotin of *K. obovata*. Our study provided valuable clues for further exploring the function of *KoOsmotin* response to stress.

## Background

As an important marine wetland ecosystem, mangroves was mainly distributed in tropic and subtropic estuaries, which usually experienced variable and complex abiotic stresses during their growth and development [1, 2]. *Kandelia obovata* is the most cold-resistance species in mangrove plants. Studies have shown that *K. obovata* utilized diverse physiological and structural mechanisms for rapid response to multiple stresses, such as cold, salinity, drought and water logging [3, 4]. As a multifunctional protein, osmotin played as a key regulator in response to abiotic stresses [5, 6]. Therefore, among the cold stress-response genes in our previous study [7], the *osmotin* gene might be one of the most potent candidates for improving *K. obovata* stress-resistance. However, there is little literature about osmotin or osmotin-like protein (OLP) in *K. obovata* or other mangrove plants so far.

Osmotin is a member of the pathogenesis related-5 (PR-5) protein family. PR-5 generally include proteins related to thaumatin, zeamatin and osmotin, and is recognized with antifungal activity [8]. Osmotin was first isolated from tobacco cell cultures [9], and then the gene encoding osmotin was cloned [10]. As a pre-protein, osmotin was considered to be synthesized inside the vacuole with a molecular weight of 26.4 kDa, whereas the mature form was 24 kDa [11]. Osmotin generally contains sixteen conserved cysteine residues that are distributed throughout the protein and form eight disulfide bridges [12]. These conserved cysteine residues can help to stabilize the molecule, accurate folding and prevent protease degradation [13]. Osmotin is made up of three motifs (Domain I-III) that show similar folding in other PR-5 proteins such as thaumatin and zeamatin [12]. Osmotin is a secretory protein that does not contain introns, DNA-binding motifs and glycosylation locations [14], however, it generally contains a C-terminal polypeptide extension, which is necessary for localization of vacuole [15].

Although intensive studies have been conducted to the defense function of osmotin, the intricate interplay of *osmotin* in gene regulation was still unclear. Primarily, many studies hypothesized that *osmotin* might be a transcription factor to regulate key genes in response to abiotic and biotic stresses [16, 17]. However, later evidence had ruled out this hypothesis by the fact that osmotin did not contain any DNA-binding motifs [14]. Besides, the osmotin was also shown to activate mitogen-activated protein kinase (MAPK), which was fundamental to most signaling and regulatory processes [18]. Consequently, researchers recently suggested that osmotin was the key regulator mediating plant in response to various stresses [19]. Furthermore, osmotin played essential roles in protecting native protein structures and repairing denatured proteins [20]. Under salinity and drought stresses, osmotin showed the ability of maintaining cellular osmolarity by compartmentalization of solutes or by structural and metabolic changes [21]. In *Petunia hybrida*, osmotin was induced by several apparently unrelated environmental signals, illustrating its essential complexities of gene expression [22]. Osmotins have been characterized from diverse plant species and were induced by biotic and abiotic stresses [22–24]. Over-expression of *osmotin* genes in transgenic plants can enhance tolerance against stresses including cold, drought, high-salinity and some combinations of them [19, 25–27]. In olive plant, overexpression of osmotin showed multiple effects on Ca^2+^ signaling, cytoskeleton dynamics and programmed cell death [28]. In addition, combined overexpression of *osmotin* and *chitinase* genes has enhanced antifungal activity in transgenic rice [29]. Researches also supported that osmotin played orchestrated activities with other cold-related genes in plants [25, 30].

A partial nucleotide sequence (Ko3113) showing high homology with *osmotin* was isolated from the cDNA library of *K. obovata* in our previous study [7]. Here, we further cloned and characterized the full-length of this gene, and named it *KoOsmotin.* The expression patterns of the gene in plant systems (roots, stems and leaves) under cold stress were performed. The subcellular localization of KoOsmotin was determined by expressing green fluorescent protein (GFP)-tagged *KoOsmotin* in transgenic tobacco plant*.* Furthermore, function analysis of cold tolerance was carried out by overexpressing *KoOsmotin* in *E. coli* cells. This study will provide a good start for *KoOsmotin* in stress defense in *K. obovata*, and also help to improve the mechanism of stress resistance in mangrove plants.

## Results

### Isolation and structural analysis of the *KoOsmotin* from *K.obovata*

**Fig. 1:**
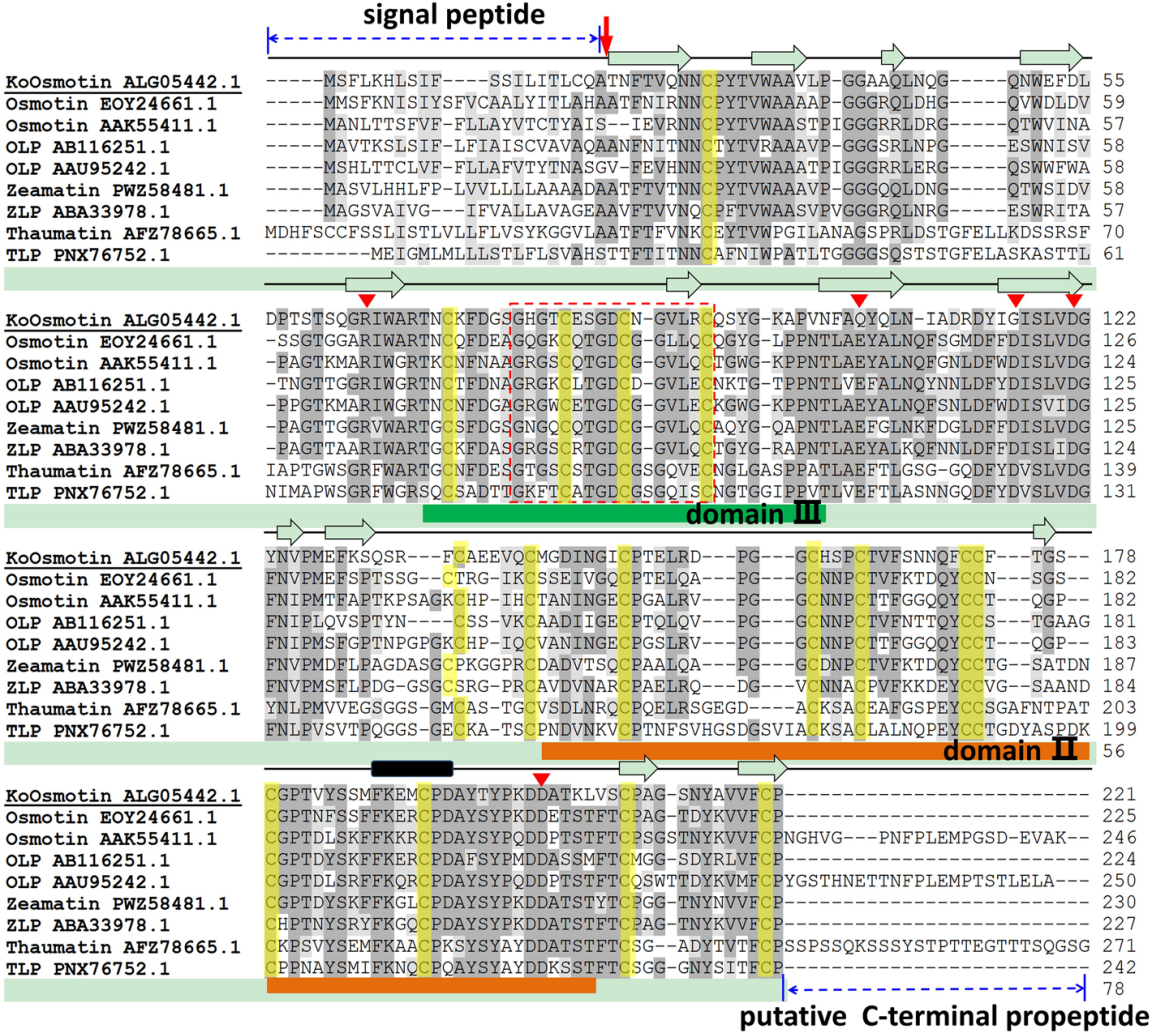
Sequence alignment of KoOsmotin and other well-studied plant PR-5 proteins. Only the complete published sequences were used. Identical and similar amino acids among PR-5 proteins were colored dark and light gray, respectively. A cleavage site in KoOsmotin was indicated by a red arrowhead on the N-terminus. Predicted *α*-helix and *β*-strand were indicated above the sequences in dark boxes and in white wide arrows, respectively. Conserved positions containing five amino acids of the central cleft were labeled by red triangles. The sixteen conserved cysteine residues, possibly involved in the formation of disulfide-bridges in PR-5 proteins, were highlighted by yellow shadows. The N- and C-terminal elongations, predicted as signal peptide responsible for extracellular secretion and for vacuolar location, respectively, were indicated with blue lines. Sequences boxed in red dotted line showed the thaumatin motif G-x-G/F-x-C-x-T/S-G/A-D-C-x-G/Q-x-x-x-C, which was family signature in PR-5 proteins. The domain II and domain III in the 3D structures were indicated by green and orange boxes under the sequences, respectively

**Fig. 2:**
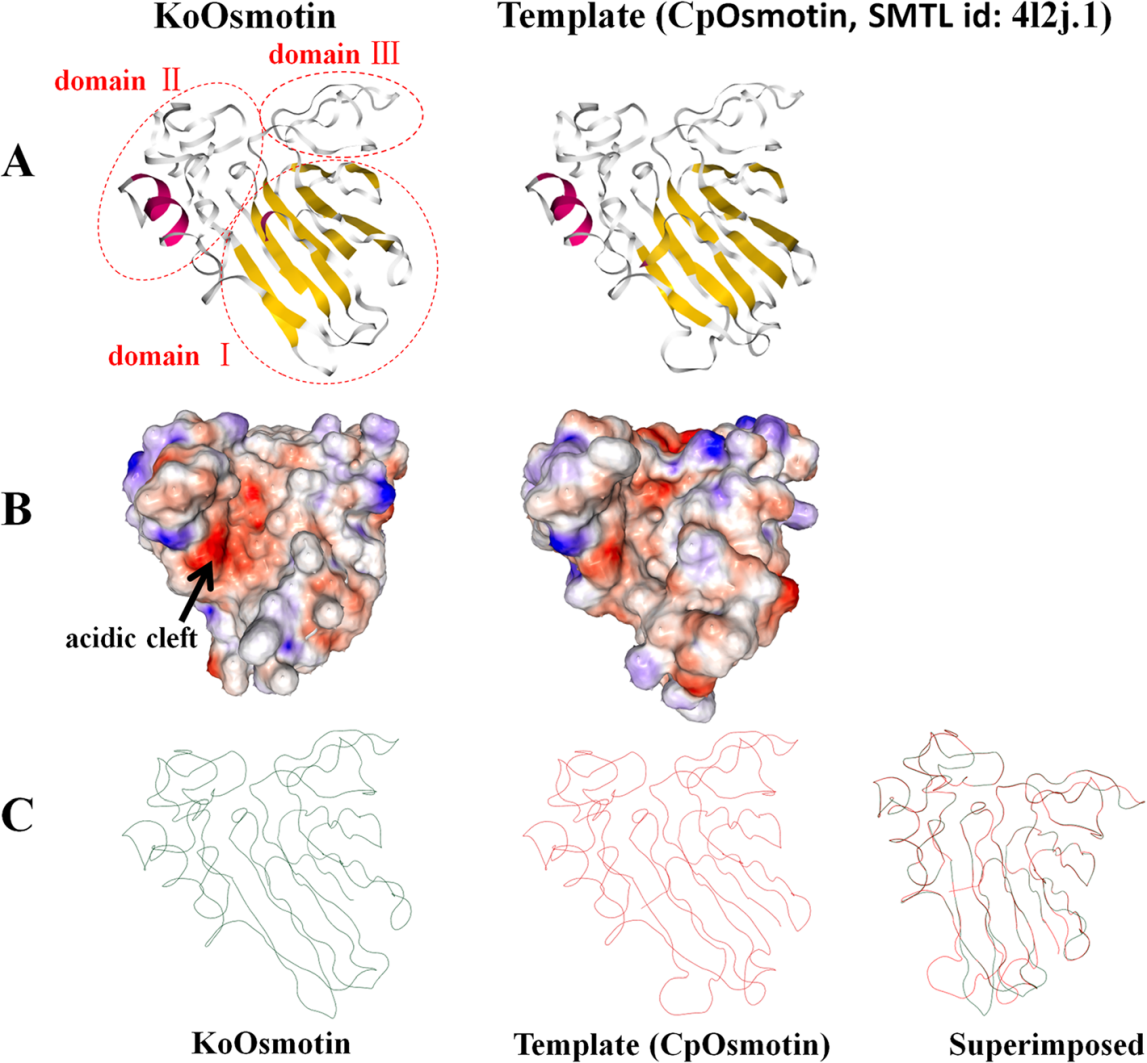
Prediction of 3D structure of KoOsmotin and comparison with its template CpOsmotin. The 3D models of KoOsmotin and its template (CpOsmotin, SMTL id: 4l2j.1) were produced by the homology modeling SWISS-MODEL. **A**. Pictures are predicted ribbon structures of KoOsmotin and its template, with *α*-helix (red) and *β*-strand (yellow), random coil (white). Three domains of KoOsmotin were indicated by red ellipses (domain I, amino acids1–68,102–142, 207–221. domain II, amino acids 143–206. domain III, amino acids 69–101). The three domains were similar in the 3D topology with other PR-5 proteins. **B**. View of the surface topology of KoOsmotin (left) and its template (right) showing the distribution of the electrostatic potentials. Protein surface was colored according to areas charged positively (blue), negatively (red) and neutrally (white). The acidic cleft was indicated by a black arrowhead. **C**. Figures are predicted trace structures of KoOsmotin and CpOsmotin and their superimposition

An osmotin cDNA sequence from *K.obovata*, designated as *KoOsmotin* (GenBank accession no. KP267758), was isolated by SMART™ RACE cDNA amplification. The nucleotide sequence of *KoOsmotin* was 1126 bp, which contained a 666 bp open reading frame (ORF) encoding a deduced protein length of 221 amino acids. The ORF started at the ATG initiation codon at position 196, and terminated at the TAG terminator codon at position 861. The calculated molecular weight and p*I* of the putative KoOsmotin protein were 24.11 kDa and 4.92, respectively. The amino acid composition analysis showed that KoOsmotin contained high content of Gly (8.6%), Ser (8.6%), Cys (7.7%), Ala (6.3%), Thr (6.3%), Asn (6.3%), and Val (5.9%), while that Trp occupied the lowest (1.4%) portion. Secondary structure analysis indicated that the KoOsmotin included 9.05% *α*-helix, 28.96% extended strand, 4.07% *β*-turn and 57.92% random coil (distributed details were shown in Fig. 1). The KoOsmotin included 19 negatively charged residues (Asp and Glu) and 13 positively charged residues (Arg and Lys), which was accordant with negatively charged on most surface of KoOsmotin (Fig. 2B). Besides, predicted grand average of hydropathicity (GRAVY) of KoOsmotin was − 0.158. Since a negative GRAVY value always showed the hydrophilic nature of proteins, indicating KoOsmotin was a hydrophilic prtoein. TMpred prediction analysis showed that KoOsmotin had a transmembrane helix (residues 17–33). The KoOsmotin also contained two conserved residues (Asp121, and Asp202), which will form acidic cleft areas in the structure (Fig. 2B). In addtion, KoOsmotin has the conserved characters of an osmotin, such as no clear glycosylation locations, no introns, no DNA-binding motifs and no allergenic motifs. The results have been consistent with previous reports about osmotin [14].

The BLASTx search on the NCBI database indicated that the KoOsmotin showed the greatest similarity to osmotin 34 (GenBank accession no. EOY24661.1) from *Theobroma cacao* [31], with 55.75% amino acid identity in 100% coverage. The BLAST search aslo revealed that the amino acid sequence of KoOsmotin had high similarity to some PR-5 proteins, such as thaumatin and thaumatin-like proein (TLP). Thus, a multiple sequences alignment of amino acids sequences was performed to compare KoOsmotin with other well-studied PR-5 proteins, including osmotin [22, 31], osmotin-like protein (OLP) [32, 33], zeamatin [34], zeamatin-like protein (ZLP) [35], thaumatin [36] and TLP [37]. Although the alignment of KoOsmotin with these eight well-characterized PR-5 proteins revealed similarity up to 52.9%, the KoOsmotin contained the characteristic sequences of osmotin or PR-5 proteins (Fig. 1). Motif Scan analysis showed that KoOsmotin contained a very conserved typical motif (residues 27–221) that belonged to thaumatin family. A special fingerprint (residues 77–92), G-x-G/F-x-C-x-T/S-G/A-D-C-x-G/Q-x-x-x-C, so-called namely thaumatin motif, was found in KoOsmotin (Fig. 1). This thaumatin motif were highly conserved in osmotins and other PR-5 proteins [38, 39]. Moreover, KoOsmotin contained sixteen cysteine residues and formed eight disulfide bridges. The disulfide bridges were deemed to assist the stabilization of protein molecules and allows for accurate folding [12]. All the sixteen cysteine residues present in KoOsmotin were highly conserved in osmotin and other PR-5 proteins (Fig. 1). Similar to these proteins, KoOsmotin included a signal peptide for secretion near the N-terminus (residues 1–20) and a cleavage site (Fig. 1). Although a C-terminal domain is always present in osmotin and PR-5 proteins [40], the KoOsmotin lacked C-terminal polypeptide, which was also absent in some osmotins and other PR-5 proteins (Fig. 1). From these results, the KoOsmotin was identified as a member of osmotins, belonging to the PR-5 proteins family, which was involved in plant defense against pathogens [41].

### Three-dimensional models of *KoOsmotin*

Tertiary structures of KoOsmotin was predicted by homology modeling SWISS-MODEL [42]. The KoOsmotin showed the highest model homology (58.08%) with the template (Osmotin: antifungal laticifer protein, SMTL id: 4l2j.1.A), CpOsmotin, from *Calotropis procera* [43], and other TLPs (data not shown). Since formation of 3D model requires 50% similarity between compared principle amino acids sequences [44], indicating the 3D model of KoOsmotin was reasonable and receivable. The homology modeling revealed that the structure of KoOsmotin was composed of three domains, which were common in PR-5 proteins. The 3D model of KoOsmotin contained one *α*-helix, eleven *β*-strands and some random coils, which further formed three characteristic domains: “domain I” (residues 1–68, 102–142, 207–221) included eleven *β*-strands, in the form of a compacted sandwich, “domain II” (residues 143–206) contained one *α*-helix and “domain III” (residues 69–101) comprised two single loops (Fig. 2A). Domains I and II form an acidic cleft in the KoOsmotin (Fig. 2B). Study reported that many variations were presented in domain-II among osmotins [8]. However, the obvious difference between KoOsmotin and its template CpOsmotin was domain I. The skeletal differences showed variability and were found as altered loop orientations (residues 109–112, 132–137) in domain I (Fig. 2C). As an acidic PR-5 protein, KoOsmotin had widespread negative areas, in particular, the molecular surface of KoOsmotin was almost charged negatively. The distribution of the electrostatic potentials of KoOsmotin was obviously different from its template CpOsmotin. (Fig. 2B). These differences in the skeletal basis and in the surface electrostatic potential, possibly involved in stress-resistance, might be decisive for the specific interaction and activities between PR-5 proteins.

### Phylogenetic relationship of *KoOsmotin*

**Fig. 3:**
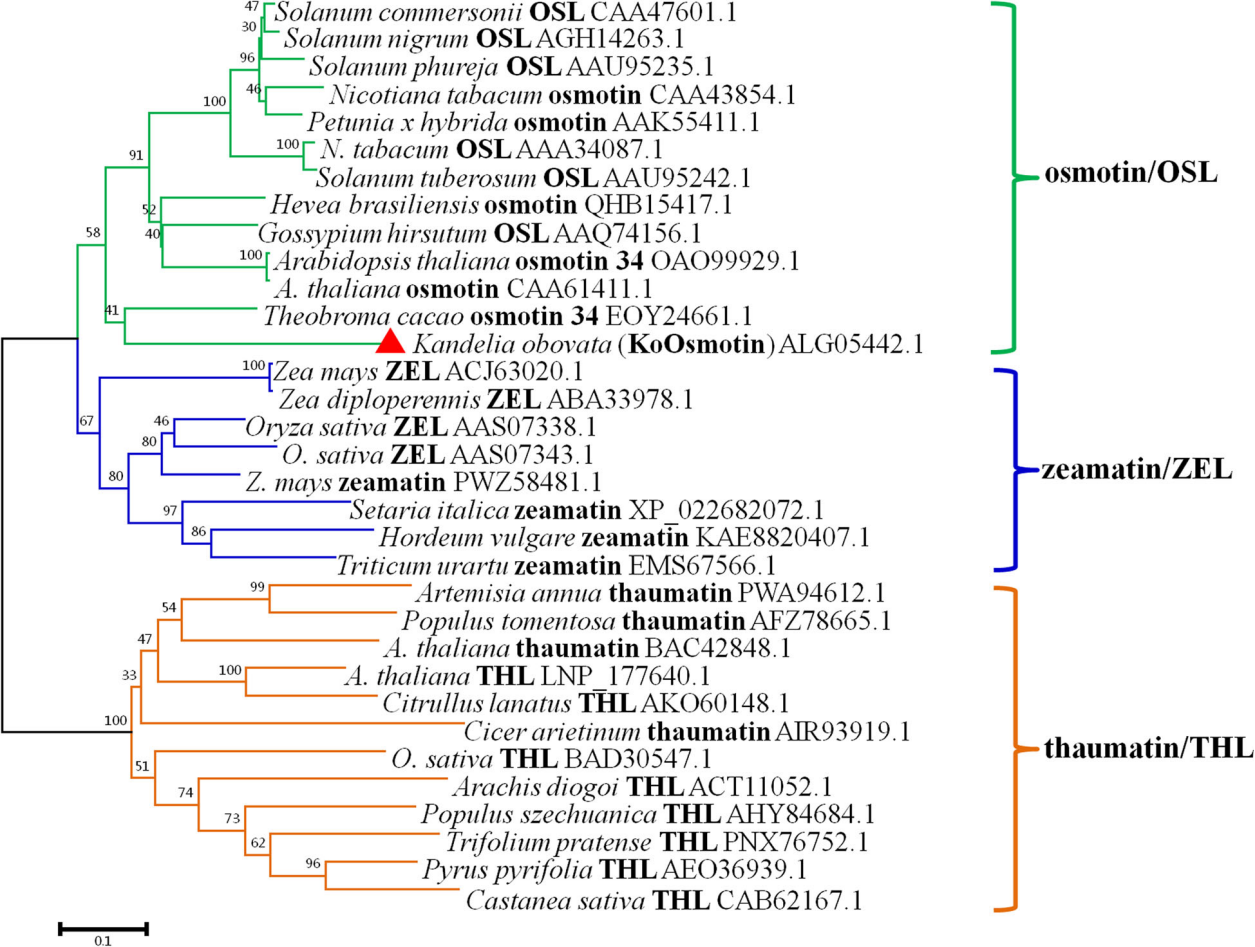
Phylogenetic relationship between KoOsmotin and other plant PR-5 proteins. The entire tree including 33 amino acid sequences, which were obtained from the NCBI database with accession number indicated. The red trilateral indicated the KoOsmotin. This tree was constructed by the neighbor-joining method with 1000 bootstrap replication. The scale indicated the branch length

Osmotins have been characterized in many plants. Thus, many partial and complete sequences of osmotins are available in the NCBI database. The NCBI database indicated that the KoOsmotin showed homology with osmotin and some other PR-5 proteins, such as thaumatin and TLP. To clarify the evolutionary relationships of KoOsmotin with other PR-5 proteins, the phylogenetic tree was constructed. The 33 homologues of PR-5 proteins from various plants were used as the basis for the tree. The overall phylogenetic tree was consisted of three major clusters, osmotin/OLP, zeamatin/ ZLP, thaumatin/TLP, each containing proteins with relatively high identity. The cluster groups were generally well-supported by the bootstrap values. The phylogenetic tree showed that KoOsmotin had the close genetic relationship to the supported clade of osmotin/OLP, closest with osmotin 34 from *T. cacao* (Fig. 3), thus further demonstrating that the KoOsmotin was a member of osmotins. The phylogenetic analysis also indicated that PR-5 proteins from the same species had considerable variations, such as *Arabidopsis thaliana* and *Oryza sativa*. The evolutionary analysis indicated that the PR-5 protein family was highly divergent, which confirmed *PR-5* gene family was an ancient multigene family conserved in plants [45].

### Subcellular localization of *KoOsmotin* in tobacco epidermal cells

**Fig. 4:**
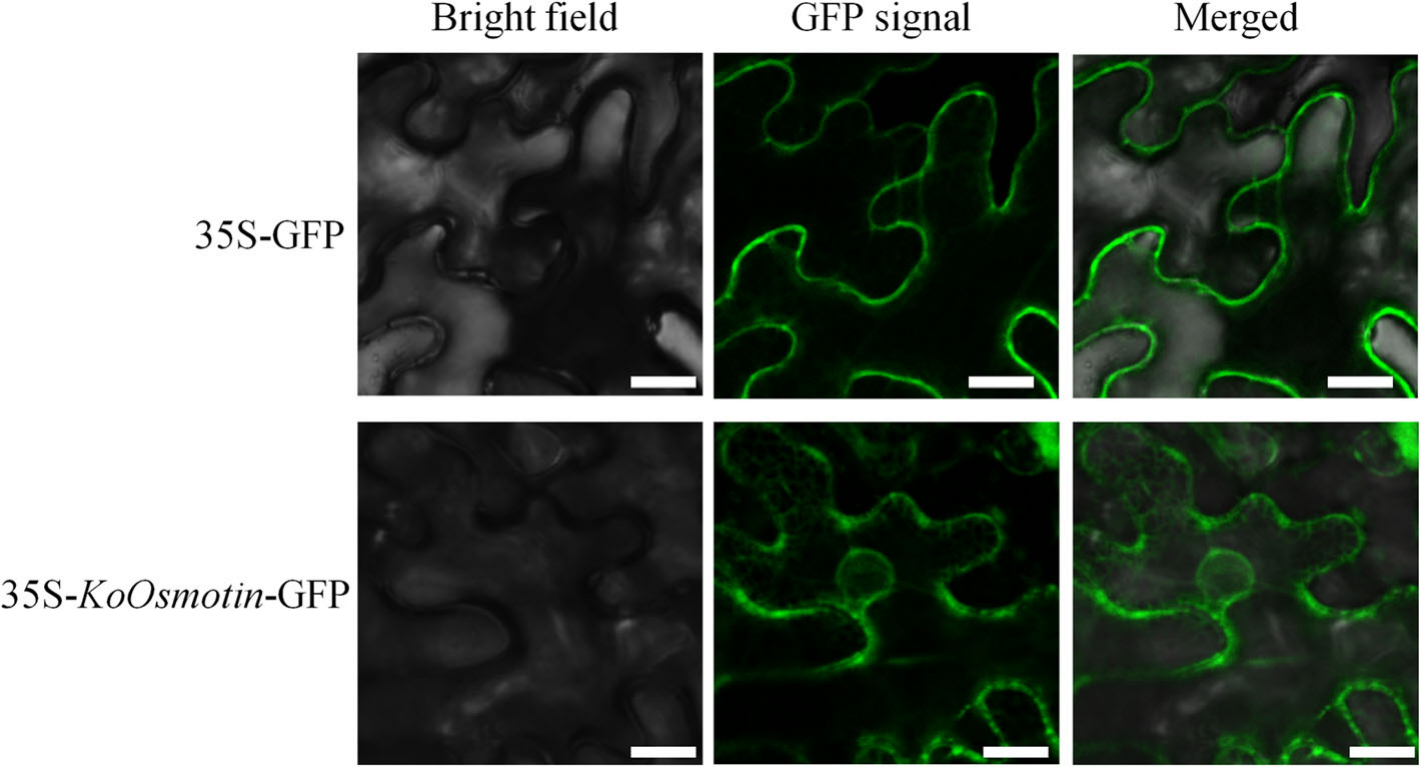
Subcellular localization of KoOsmotin in *N. benthamiana* epidermal cells. Transient KoOsmotin expression was visualized by GFP fluorescence. The leaf tissue overexpressing 35S-KoOsmotin-GFP and only 35S-GFP (control) were imaged by confocal microscop. The bar in the lower right corner of these images indicates 10 μm

According to the online prediction tools, Cell-Ploc 2.0 and Softberry, the KoOsmotin was predicted to be localized to cytoplasm and vacuole, respectively. To further validate the subcellular localization of KoOsmotin in plants, the fluorescence-tagged 35S-*KoOsmotin*-GFP was generated and transformed into *N. benthamiana* epidermis cells. As shown in Fig. 4, the fluorescence signal of 35S-KoOsmotin-GFP was accumulated heavily around the cell borders with spotted distribution, and diffused weakly in cytoplasm of the host plant cells. As a secretory and mature protein, the KoOsmotin presented in the cytoplasm might be secreted from vacuole. However, in *N. benthamiana* epidermal cells whereas expressing only the empty vector, the fluorescence was observed exclusively on the plasma membrane with a linear distribution, and none of them was localized to cytoplasm (Fig. 4). These results indicated that the KoOsmotin was localized to both the plasma membrane and cytoplasm where they might fulfill different functions. Osmotin has also been confirmed to be localized to the plasma membrane in previous study [19].

### Gene expression of *KoOsmotin* in *K. obovata* induced by cold stress

**Fig. 5:**
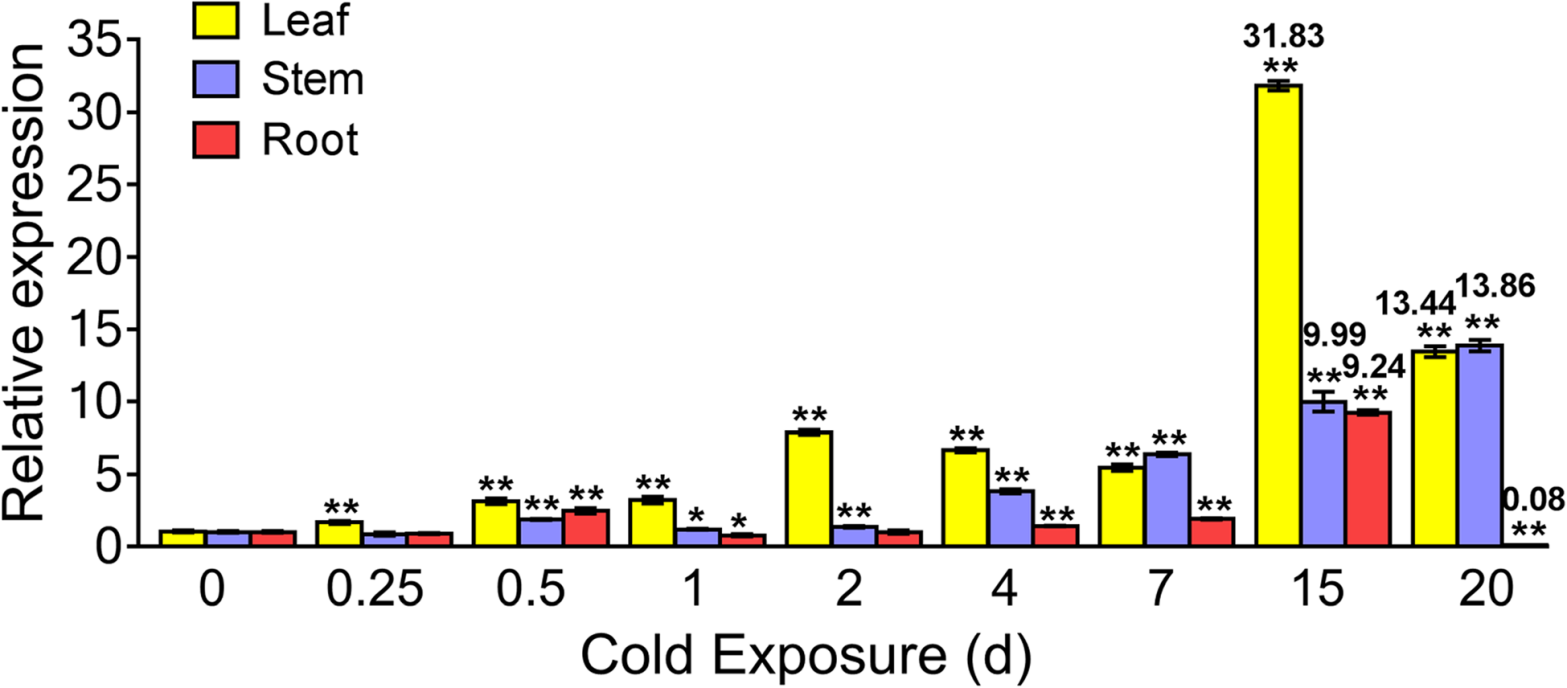
Expression analysis of the *KoOsmotin* transcripts in *K. obovata* under cold stress. Types of tissues were indicated. The relative expression levels of *KoOsmotin* were normalized by the expression of reference gene, *Ko18S*. Values represented means of three biological replicates. Error bars indicated the standard deviations (*p* values and were calculated based on Student’s t test. *p < 0.05; **p < 0.01; ***p < 0.001)

To determine the expression patterns of *KoOsmotin* induced by cold stress in whole plant, the levels of *KoOsmotin* transcripts in leaves, stems and roots were examined. Total RNA was isolated from various tissues of *K. obovata* seedlings after cold stimulation. The real-time quantitative PCR (RT-qPCR) results revealed that gene expression of *KoOsmotin* was induced highly in the leaves under cold stress, but lower in the stems and roots (Fig. 5). Although the *KoOsmotin* gene responded positively or negatively at some time points, the expression patterns of *KoOsmotin* showed similar expression tendency in leaves and roots. In leaves, the expressions of *KoOsmotin* were induced after the initiation of cold stimulation and stimulated to the top (31.83-fold) at 15 d, but sharply decreased at 20 d. Similar tendency was present in roots. The highest expression level (9.24-fold) of *KoOsmotin* was also observed at 15 d, and rapidly induced at 20 d in roots. The difference between them reflected that the expression level at 20 d was still much higher (13.44-fold) than the control (0 d) in the leaf, but in root, the level was much lower (0.08-fold) than its control. Interestingly, in the stems, the amount of *KoOsmotin* transcripts was progressively increased with the extension time after cold stimulation, and reached the peak (13.86-fold) at 20 d. Noticeably, the amounts of *KoOsmotin* expression were all induced to a small extent at first 7 d, but sharply increased to substantial accumulation after 15 d among in both leaves, stems and roots, respectively. These results indicated that the *KoOsmotin* responded positively to cold stress at late phases in different tissues, especially in leaves, where photosynthesis takes place. This implied that *KoOsmotin* gene might be an important part of the signaling networks that *K. obovata* replying to cold stress.

### Overexpression of *KoOsmotin* enhances cold tolerance in transformed *E. coli*

**Fig. 6:**
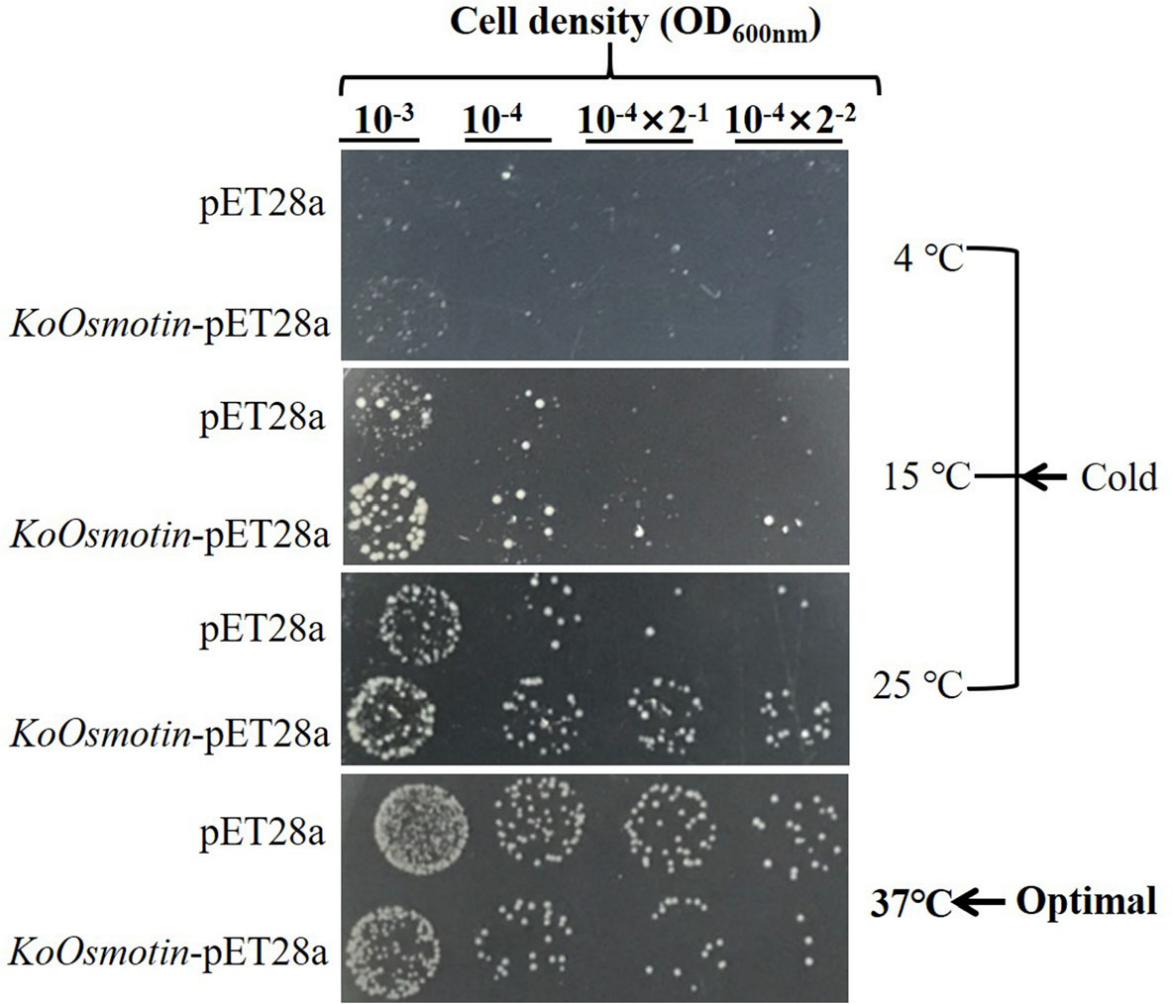
Cold tolerance analysis of *E. coli* transformed with *KoOsmotin*. The *E. coli* cells was spotted onto LB agar medium. Since the optimal cultured temperature for *E. coli* was 37 ℃, the cold treatments were performed under 5, 15 and 25 °C, respectively. The four concentration gradients of *E. coli* cell suspension were shown above the figure

To investigate the functional role of *KoOsmotin* in cold stress tolerance, the *KoOsmotin* was cloned and overexpressed in *E. coli*. Transformed *E. coli* cells with *KoOsmotin*-pET28a were generated to test the cold-resistance of *KoOsmotin*. Under optimum condition (37 °C), the *E. coli* cells transformed with *KoOsmotin*-pET28a showed slightly weaker growth than the control (Fig. 6). However, among all the different cold treatments (5 °C, 15 °C, 25 °C), the *E. coli* cells transformed with *KoOsmotin*-pET28a exhibited significantly enhanced growth when compared with the control (Fig. 6). These results indicated that overexpressing *KoOsmotin* could confer the cold resistance for *E. coli* and help to increase the growth of *E. coli* under cold stress. Previous studies have proved that overexpressing *osmotin* gene could help to enhance cold tolerance in transgenic plants [19, 27]. Thus, we could infer that overexpressing *KoOsmotin* might confer cold-resistance in transgenic plants. Using *E. coli* or other microorganism to analyze the function of plant genes had been adapted in previous studies [46–49]. In this study, the functional analysis of *KoOsmotin* using *E. coli* might be used to predict its tolerance in transgenic plants. This is only the start for the function of the *KoOsmotin* in cold-resistance, further investigations are needed to be performed.

## Discussion

In this study, a cold-inducible *osmotin* gene encoding an acidic PR-5 protein, designated as *KoOsmotin*, was identified from *K. obovata*. We characterized this gene at structural and transcriptional levels, as well as its subcellular localization. Furthermore, we verified the cold resistance of *KoOsmotin* in *E. coli* cells. Usually, the tertiary structure of osmotin was homologous to thaumatin, zeamatin and TLP [45]. Consistent with this, the KoOsmotin showed the highest similarity with osmotin, followed with thaumatin or TLP according to the NCBI database and Swiss Model search. Generally, osmotin and thaumatin resembled each other on the basis of their structure, molecular weight and their conserved disulfide bonds. Besides, osmotin was made up of three domains that shows similar folding with thaumatin and zeamatin [12]. The result of phylogenetic tree in this study further confirmed the close relationship among these three proteins. However, the thaumatin protein tasted sweet, whereas osmotin does not have a sweet taste [8].

In this study, the predicted molecular weight of KoOsmotin was 24.11 kDa, which consistent with precious study [11], indicating KoOsmotin was a mature protein. A C-terminal domain is always supposed to be present in osmotin and PR-5 proteins [40]. Nevertheless, the sequence of KoOsmotin lacked the C-terminal polypeptide, which was also absent in some other osmotins, originally purified as a mature protein from the healthy leaves [31]. Some other PR-5 proteins similar with no C-terminal elongation were also found in zeamatin [34], ZLP [35, 50, 51], TLP [38, 52] and OLP [33]. Plant osmotins have been reported to be localized on different cellular compartments, including plasma membrane, vacuole, chloroplast and endoplasmic reticulum [19]. The bioinformatic tools predicted that KoOsmotin was localized to cytoplasm or vacuole or extracellular. Further evidence performed by confocal microscope analysis revealed that KoOsmotin was mainly localized to plasma membrane, which was consistent with osmotin, TlOsm, from *Tripogon loliiformis* [19]*.* However, the TlOsm possessed a C-terminal elongation, which was always considered to be necessary for vacuolar targeting [40]. Besides, many PR-5 proteins lacking the C-terminal elongation but with the N-terminal signal peptide only, like KoOsmotin here, were generally thought to be secreted into the extracellular matrix [33, 50], and were localized in the apoplastic space of plant [38]. The truth is that the studies performed for subcellular localization by confocal microscope showed different phenomena. Thus, an osmotin may concurrently localize to multiple cellular compartments. TMpred analysis predicted a transmembrane fragment in KoOsmotin, suggesting this region was responsible for the plasma membrane localization of the KoOsmotin. It is always accepted that plasma membrane generally contains proteins that are fundamental for stress-signal perception and signal transduction into downstream genes. Previous studies showed that *osmotin* gene was likely to be upstream gene and involved in stress signal transduction [14, 22, 30, 40, 53]. This suggested that the KoOsmotin may function as stress-responder on plasma membrane and play a key role in regulating downstream genes under stress.

KoOsmotin model contained an acidic cleft between the domains I and II. This acidic cleft was supposed to determine PR-protein specificity to their target receptors or ligands for an antifungal activity, whereas no antifungal thaumatin had a basic cleft [12, 45]. The acidic cleft regions of KoOsmotin were essential for antifungal activity because of five amino residues (Arg^64^, Gln^104^, Gly^116^, Asp^121^ and Asp^202^) (Fig. 1). These amino residues were assumed to be responsible for topology and surface electrostatic potential around the cleft. In KoOsmotin, three of five acidic residues (Arg^64^, Asp^121^ and Asp^202^) were conserved in other PR-5 proteins. The other two neutral and hydrophilic residues (Gln^104^ and Gly^116^) of KoOsmotin were replaced by Glu and Asp, respectively, which existed in most PR-5 proteins. The two acidic residues (Glu and Asp) were also replaced in a few PR-5 proteins [13]. In *K. obovata*, the two amino acids (Gln^104^ and Gly^116^) of KoOsmotin were different from most plant PR-5 proteins, might indicating the special stress-adaption to tidal area environment that *K.obovata* growing up. As a secreted protein, the results implied that KoOsmotin might possess special antifungal activity, which help *K.obovata* plant to grow in the harsh natural habitats.

The RT-qPCR results revealed that gene expression of *KoOsmotin* was highly induced in the leaves, but lower in the stems and roots. Besides, the expression patterns of *KoOsmotin* were not synchronized in the roots and stems. It was clear that the expression of *KoOsmotin* exhibited tissue-specific expression, which were also observed in other *osmotin/OLP* genes. In *Petunia hybrida*, the *osmotin* amounts were high accumulated in roots but slightly in stems and leaves [22]. The tobacco *osmotin* was mainly induced in flower, but not in shoots after wounding [54]. In the case of soybean, the *GmOLPb* gene was highly induced in leaves but lower in the stem by methyl jasmonate stimulation [38]. Previous studies showed that osmotin has the ability to protect chlorophyll and photosynthetic machinery, prevent reactive oxygen species accumulation and stimulate more proline accumulation [5, 16, 55]. The amounts of *KoOsmotin* expression were all induced to a small extent at first 7 d, and the expression levels in leaves were higher than in stems and roots. Thus, it can be concluded that KoOsmotin would be released into the extracellular space and then function in the defensive systems to protect *K. obovata* cells (mainly in leaves) in the early phase of cold stress. Cold stress is usually accompanied by dehydration and osmotic imbalance with time extending. Studies have certified that osmotin had a function in osmoregulation under stress and had adapted to low osmotic potential environments [14, 24, 56]. The expression levels of *KoOsmotin* were increased to substantial accumulation after 15 d under cold stress in among leaves, stems and roots. Therefore, it came to infer that *KoOsmotin* might function to accumulate abundantly in the late phase of cold treatment to protect *K. obovata* against the imbalance and impart tolerance to cold stress.

In addition, it was worth mention that the expressions of *KoOsmotin* were increased highly at 15 d under cold stress in both leaves and roots. However, their expressions were both decreased rapidly at 20 d, specially very low (0.08-fold) compared with its control in root. The changes of *KoOsmotin* expressions were generally consistent with morphological changes. The seedlings treated at cold treatment (5 °C) for 15 d were cultivated under recovery-temperature (25 °C) for 20 d, all these seedlings were still alive. However, at the same recovery condition, the seedlings treated at cold treatment (5 °C) for 20 d were cultivated, all the leaves of these seedlings were withered and fade (data was not shown here). This might indicate that the expression of *KoOsmotin* had a close relationship with the *K.obovata* survival and played a key role in protecting *K. obovata* against cold stress. According to the high consistency between *KoOsmotin* expression on molecular level and recovery survival on morphological level, it can be deduced that 15–20 days maybe the time-limited range for *K. obovata* seedlings under cold treatment (5 °C). This will provide reference for north introduction of *K. obovata*.

Many osmotins had been identified and characterized, and possessed the ability to enhance cold resistance by overexpression in transgenic plants [19, 27, 28]. Overexpressing tobacco osmotin gene in olive plant revealed that the *osmotin* gene could prevent cold-induced calcium signaling, regulate the dynamics of the cytoskeleton and cold-related programmed cell death [57]. Moreover, the overexpression of the *osmotin* gene in tobacco could stimulate the expression of downstream genes that were involved in proline biosynthesis under cold conditions [16]. Our study also showed an enhanced cold resistance by overexpressing *KoOsmotin* gene in transformed *E. coli* cells. Use prokaryotes to analysis the function of plant genes had been adapted in many studies [46–49]. Although further investigations are needed, we can infere that *KoOsmotin* might also possess cold-resistance in transgenic plants. Besides, many scientists have successfully transformed *osmotin* gene to induce salt, drought and osmotic stress resistance in transgenic plants [16, 19, 26, 55, 57, 58], as well as antifungal activity [40, 59, 60]. These literatures lead us to the conclusion that osmotin is an important PR-protein and is expected to be successfully used in developing plant defense mechanisms in the future. Therefore, it is a great need to further explore the function of this important protein KoOsmotin under biotic and abiotic stress conditions.

## Conclusions

This is the first study to explore the osmotin of *K. obovata*. It also provided valuable clues for further exploring the function of *KoOsmotin* response to stress. In this study, the *KoOsmotin* was cloned and characterized from *K. obovata*. Besides, overexpressing *KoOsmotin* enhanced cold resistance and increased the growth in *E. coli.* Further studies, including analyses of its functions using transgenic plants and recombinant proteins, will reveal the exact roles and functions of *KoOsmotin* involved in biotic and abiotic stress tolerance.

## Methods

### Plant material and treatments

The hypocotyls of *K. obovata* were purchased from Guangdong Mangrove Ecological Technology Co. LTD (China). The hypocotyls were germinated in clean sands, and watered with the 1/2 Hoagland solution. At the four-leaf stage, the seedlings were transferred to a growth chamber (25 °C, 75% humidity, 14 h light/10 h dark cycle). The seedlings were cultivated under cold stress (5 °C) for 0, 0.25, 0.5, 1, 2, 4, 7, 15 and 20 d (day), respectively. All the treatments contained at least three seedlings. The samples (leaves, stems and roots) were washed by ultrapure water and dried by clean paper towels before collected. The samples collected at 0 d were used as the controls. All the harvested samples were immediately frozen in liquid nitrogen, and stored at − 80 °C before use.

### RNA isolation and cDNA synthesis

Total RNA was extracted using Plant Total RNA Extraction Kit (BioTeke Corporation, China), following the manufacturer’s protocol. The RNA pellet was dissolved in RNase free water. RNA was quantified using Nanodrop 1000 spectrophotomete (Thermo Scientific, Wilmington, DE, USA) and checked by agarose gel electrophoresis (1%). Genomic DNA in the total RNA samples was eliminated by RNase-free DNaseI (Promega, USA) following the manufacturer’s instructions. First strand cDNA was synthesized using SMART™ reverse transcription Kit (Clontech), following the manufacturer’s instructions. The 3′- and 5′-terminal sequences of *KoOsmotin* were cloned referring to the sequenced region by SMART™ RACE cDNA Amplification Kit (Clontech, USA).

### Cloning the full-length cDNA of *KoOsmotin* gene

The partial nucleotide sequence (GenBank accession no. Ko3113) of the *KoOsmotin* gene was used as the reference sequence for designing gene primers. The 3′- and 5′-terminal sequences of *KoOsmotin* were cloned by SMART™ RACE cDNA Amplification Kit (Clontech, USA) according to the manufacturer’s instruction. The gene specific primers (GSPs or NGSPs) were synthesised to carry out 3′- and 5′-RACE. GSP1 (5′- TTGTTGCTTCACTGGCAGCTGTGGGCCT-3′) and GSP2 (5′- GAACACAACCGCATAATTAGACCCGGCAG − 3′) were used as primary PCR to obtain 5′ and 3′ end sequences of *KoOsmotin* gene. NGSP1 (5′-CTGGCAGCTGTGGGCCTACTGTTTACTC-3′) and NGSP2 (5′- AACCGCATAATTAGACCCGGCAGGACAAG-3′) were used to perform nested PCR. The 3′- and 5′- RACE products were purified by agarose gel and transformed with pMD T19 vector (Takara, Japan) into *E. coli* DH5*α* competent cells. Positive clones were selected and confirmed by nucleotide sequencing. The obtained 3′- and 5′- nucleotide sequences were assembled with overlap for the full-length of *KoOsmotin* by the DNAMAN software. The assembled sequence was used to design the primers for cloning the full length of *KoOsmotin.* The new sequence of *KoOsmotin* was sent to nucleotide sequencing. Thus, the complete full-length cDNA sequence of *KoOsmotin* was confirmed and submitted to GenBank under the accession number KP267758.

### Bioinformatic analysis

The possible ORF of *KoOsmotin* was predicted by ORF Finder (http://www.ncbi.nlm.nih.gov/gorf/gorf.html). ExPASy ProtParam tool (https://web.expasy.org/protparam/) was used to predict the molecular weight, theoretical p*I* and hydrophilia. The trans-membrane domain was predicted by TMpred (http://www.ch.embnet.org/software/TMPRED_form.html). The motif sequences were detected using Motif Scan (http://myhits.isb-sib.ch/cgi-bin/motif_scan). The secondary structure was described by SOPMA tool (https://npsa-prabi.ibcp.fr/cgi-bin/npsa_automat.pl?page=npsa_sopma.html). SignalP-5.0 Server (http://www.cbs.dtu.dk/services/SignalP/) was used to predict potential signal peptide cleavage site. Cell-Ploc 2.0 (http://www.csbio.sjtu.edu.cn/bioinf/Cell-PLoc-2/) and Softberry (http://linux1.softberry.com/) were combined to predict subcellular localization. Sequence comparisons with known sequences were performed by NCBI Databases (https://www.ncbi.nlm.nih.gov/). Phylogenetic analysis was conducted by MEGA 5.0 software. Automated 3D structure building was accomplished by SWISS-MODEL tool (https://swissmodel.expasy.org/interactive).

### Subcellular localization analysis

To confirm the subcellular localization of KoOsmotin, the entire ORF of *KoOsmotin* without the stop codon was cloned in the vector pFGC5941-35S-GFP using Hieff Clone® Plus One Step Cloning Kit (Yeasen Biotech, China) following the manufacturer’s instruction. The recombinant plasmid 35S-*KoOsmotin*-GFP was sequenced and analyzed to confirm successful fusion. The confirmed recombinant plasmid was introduced into *Agrobacterium tumefaciens* strain EHA105 and grown in a Luria-Bertani (LB) medium supplemented with kanamycin (50 μg/mL) overnight at 28 °C. *A. tumefaciens* suspension harboring 35S-*KoOsmotin*-GFP plasmid was transiently transformed in *Nicotiana benthamiana* leaf, with only pFGC5941-35S-GFP vector as the control. The GFP fluorescence signal in the leaf epidermal cells of *N. benthamiana* was imaged by a Zeiss LSM710 laser scanning confocal microscope. A × 63 oil immersion objective was used for confocal imaging. For excitation of fluorescence proteins, the 488 nm line from the argon ion laser was used to capture fluorescence. Excitation and emission wavelengths were 489 nm and 510 nm for GFP signal detection, respectively.

### Expression analysis by RT-qPCR

To investigate transcription levels of *KoOsmotin* under cold stress, the RT-qPCR method was used to determine the levels in leaves, stems and roots of *K.obovata*. The RT-qPCR reactions were performed with iCycler iQ5 real time PCR detection system (Bio-Rad, CA, USA) using SYBR Premix Ex TaqTM II reagents (Takara, Japan) according to the manufacturer’s protocol. The specific primers of *KoOsmotin* (forward primer, CTGTGGGCCTACTGTTT, reverse primer, TTTGTGGCATCGTCTTT) were designed. The *18S rRNA* of *K. obovata* was used as the internal reference gene. The PCR protocol was as follows: 95 °C for 30 s, 40 cycles at 95 °C for 5 s, 55 °C for 30 s and 72 °C for 30 s. Each RT-qPCR reaction was performed with three replicates. The transcript expression of *KoOsmotin* gene was quantified by the 2^-△△CT^ method [61, 62]. The data was presented as the mean ± standard deviation (x ± SD). All statistical analyses were performed with student t-test using GraphPad Prism version 5.0 (GraphPad Software, San Diego, California).

### Cold tolerance analysis of *KoOmotin* in *E. coli* cells

In order to validate the function of *KoOsmotin* gene in response to cold stress, the ORF region of *KoOsmotin* without the stop codon was introduced into vector pET28a-T7-His to generate recombinant plasmid *KoOsmotin*-pET28a. The *E. coli* BL21 (DE3) cells harbouring *KoOsmotin*-pET28a were cultured and used for determining the cold tolerance of *KoOsmotin*. Since the temperature 37 °C is the optimum condition for the growth of *E. coli* cells, the transformed *E. coli* cells were cultured in LB medium at 37 °C for 12–16 h. Then re-cultured them at 1% of inoculation volume for 2–3 h until the OD_600_ was about 0.6. Added IPTG (0.1 mM) and continue to cultivate for 6–8 h until the OD_600_ was 0.8. Centrifuged and suspended the *E. coli cells* in 1 mL sterilized 0.9% saline solution. Diluted the solution with sterilized 0.9% saline solution to different concentration gradients, such as 10^− 3^, 10^− 4^, 10^− 4^ × 2^− 1^, 10^− 4^ × 2^− 2^. These different concentration solutions of bacteria were used to analyze the cold tolerance of over-expressing *KoOsmotin* gene in *E. coli* cells. For cold tolerance analysis, the transformed *E. coli* cells were cultured at 5 °C, 15 °C, 25 °C and 37 °C, respectively. *E. coli* cells containing only empty vector were used as the control.

## Data Availability

The *KoOsmotin* sequence data is available from NCBI database under accession KP267758 (https://www.ncbi.nlm.nih.gov/nuccore/KP267758.1/). All data generated or analysed during this study are included in this published article. The data generated or analysed during the current study are available from the corresponding author on reasonable request.

## Abbreviations

PR-5: Pathogenesis-related 5
OLP: Osmotin-like protien
TLP: Thaumatin-like proein
ZLP: Zeamatin-like protein
GFP: Green fluorescent protein
RT-qPCR: Real-time quantitative PCR
